# A syringe-based digital algometer with a USB interface: a low-cost alternative to commercially available devices

**DOI:** 10.3389/fpain.2025.1652241

**Published:** 2025-09-04

**Authors:** Stepan Frankevich, Aryeh Simmonds, Izhak Michaelevski, Daniel Yakubovich

**Affiliations:** ^1^ Dr. Miriam and Sheldon G. Adelson School of Medicine, Ariel University, Ariel, Israel; ^2^ Department of Molecular Biology, Faculty of Natural Sciences, Ariel University, Ariel, Israel; ^3^ Liniado Hospital, Netanya, Israel

**Keywords:** pressure algometer, pain pressure threshold, concurrent validity, pain, microcontroller

## Abstract

Quantitative pain assessment is important for effective pain management. Pain pressure threshold (PPT) and Pain Tolerance (PT) measured through pressure algometry offer valuable tools for quantitative evaluation of nociceptive stimuli. Low-cost algometers, described in literature require complex calibration and lack a digital interface, limiting real-time data acquisition and integration with electronic health record systems. In the current study, we developed a durable and accurate pressure algometer built on the base of a syringe, an Arduino microcontroller and an analog piezoelectric pressure sensor. The PPT values obtained with our device are in good correlation with data obtained utilizing commercially available digital and mechanical algometers. In addition, our device can be easily connected to a computer via a USB, allowing for convenient data storage and analysis. Our results demonstrate the accuracy and reliability of a novel algometry device constructed from readily available materials and requires minimal engineering and programming skills.

## Introduction

According to epidemiological data, as much as 30% of the population regularly experience pain (1, 2). Exposure to acute pain dramatically increases risk of developing chronic pain, disrupts recovery after trauma and limits function of affected structures (3). Painful experiences combine nociceptive signaling with additional emotional and cognitive components, making accurate assessment of pain difficult (4). To address this, several methods for objective assessment of pain have been developed (5, 6).

Algometry is one of the most widely implemented methods of pain assessment (7). The basic principle of algometry is the application of a steadily increasing nociceptive stimulus until the sensation becomes painful (Pain Threshold), or until the pain becomes too distressing (Pain Tolerance). Several modes of stimuli may be utilized, including high and low temperatures and application of pressure (8).

Pressure algometers have demonstrated excellent reliability and accuracy (9). Existing designs are typically based on calibrated springs and digital pressure sensors to calculate force applied to the patient. However, the high cost of commercially available algometers can limit their use. To address this issue, several inexpensive designs have been developed. A low-cost model based on a plastic syringe has been developed for use in an emergency response unit setting (10). Another solution has been proposed in several studies—utilization of a force gauge/hand-held dynamometer as an algometer (11, 12). These devices share their working principle with algometers and demonstrate high validity and reliability.

In this study, we propose a low-cost algometer based on a plastic syringe and a high precision analog pressure sensor. This device can provide pressure data with high temporal resolution. This data can be readily stored on PC for further analysis.

## Materials and methods

**Figure 1:**
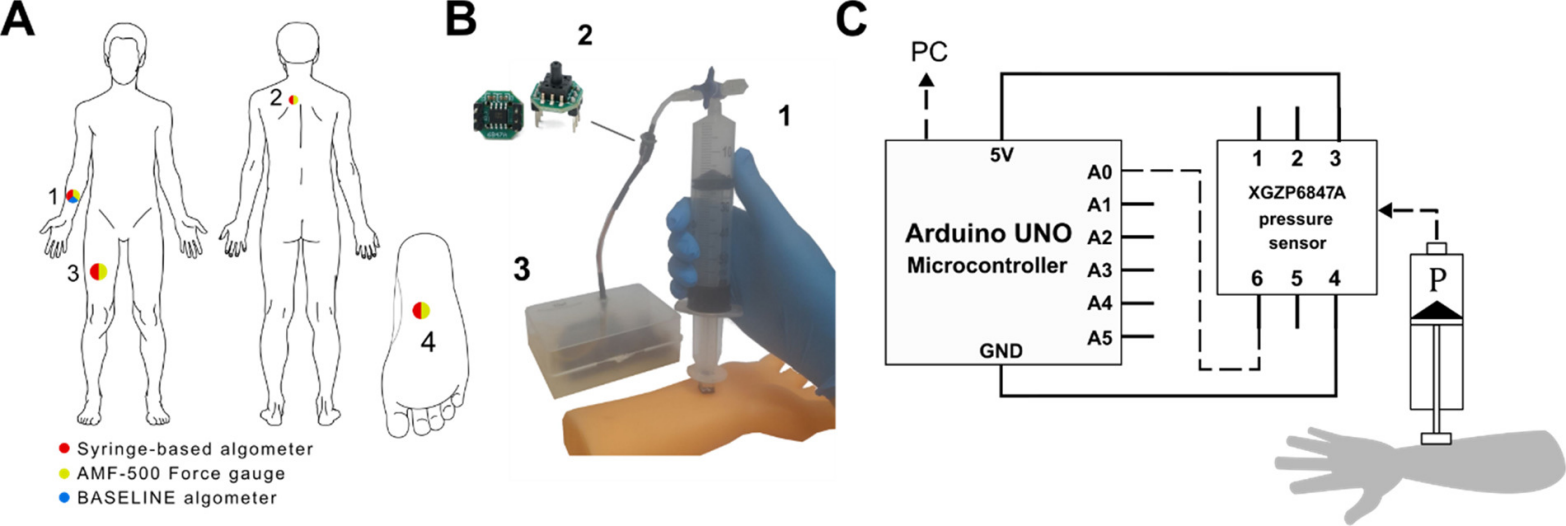
Design of the syringe-based digital algometer. **(A)** Algometer application sites: anterior midpoint of the forearm 1. the trapezius muscle 2. anterior midpoint of the thigh 3. the sole of the foot 4. Sites are color-coded, each color corresponding to relevant algometer which was tested at certain site. **(B)** Representative application of the syringe-based algometer to a model of a forearm: the air-tight syringe 1. the XGZP6847A pressure sensor 2. the Arduino microcontroller in a plastic case 3. **(C)** Schematic illustration of the syringe-based algometer. During syringe application to a specific organ air pressure increase inside the barrel (P) is continuously monitored by the pressure sensor.

The mechanism of our device is based on a piston principle similar to a previously described in literature (10). In the current study, we used plastic syringes of various volumes combined with a T-type Luer-lock valve to create an air-tight compartment. The overall design of the algometer is presented in Figure 1.

The syringe thumb rest was flattened using abrasive paper. A 1 cm^3^ acrylic cube has been affixed to the thumb rest with epoxy glue, creating a pressure probe. The side of the probe in contact with the skin has been covered with a 1 cm^2^ sheet of rubber to prevent slipping. A Luer-lock compatible T-type valve was connected to the tip of the syringe. An 85 mm long, 3 mm wide plastic tube with 1 mm-thick wall was connected to above mentioned valve. This tube served as a connector between the syringe and the pressure sensor.

Concurrent validity of pressure measurements test was done utilizing the NUL-210 pressure sensor (SES Education, Israel) instead of XGZP6847A piezoelectric pressure sensor (CFSensor, China). For validation of force measurements, we implemented the NUL-225 force plate sensor (SES Education, Israel). Nul-210 and Nul-225 sensors, which have built-in microcontrollers, were directly connected to the computer via USB. Data from these sensors was recorded and saved in the comma separated value (CSV) format using the NeuLog Windows Application (SES Education, Israel).

For data acquisition and transfer the XGZP6847A piezoelectric pressure sensor (CFSensor, China) was connected to the Arduino microcontroller (Arduino, Italy). Data recording and transfer from Arduino microcontroller to the personal computer was performed using a script written in Arduino IDE (see Supplementary Materials). Specifications of all sensors used in this work are summarized in Supplementary Table S1. For analysis we utilized pressure measurements obtained after visual stabilization of Serial monitor data output.

**Figure 2:**
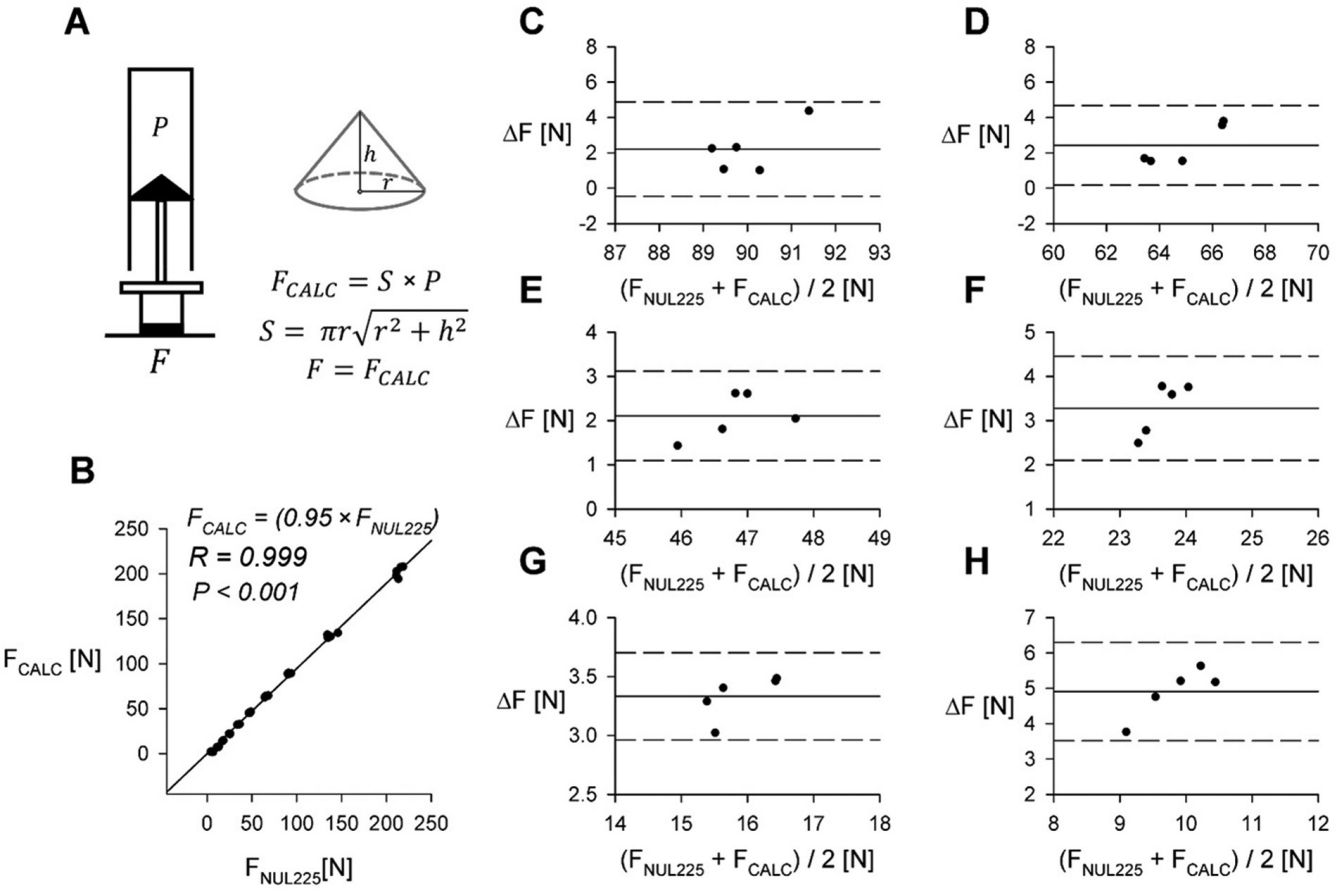
Comparison of directly measured force and calculated force. **(A)** Derivation of calculated force (F_CALC_) from P_XGZ_ and surface area of the plunger tip (S). For surface areas of specific syringe seals (see Table 1). **(B)** Comparison of applied force measured using the NUL-255 Force Plate (F_NUL225_) to force calculated from internal pressure (F_CALC_). R—Pearson correlation coefficient. **(C–H)** Bland-Altman plots comparing applied force measured using the NUL-255 Force Plate (F_NUL225_) and F_CALC_ in other barrel volumes, data from 60 ml syringe. Remaining volumes for corresponding plots: **(C)** 15 ml; **(D)** 20 ml; **(E)** 25 ml; **(F)** 35 ml; **(G)** 40 ml; **(H)** 45 ml. ΔF = F_NUL225_-F_CALC_.

To assess concurrent validity of our device we utilized the pressure values and converted them to force (see Figure 2 for explanation) which was subsequently compared to data obtained with commercially available algometers: 60 Pound BASELINE Algometer [Fabrication Enterprises, USA, similar to Fischer (12)] and the AMF-500 Digital Force gauge (ALIYIQI, China). Pain Pressure Threshold (PPT) measurements were obtained from 22 healthy volunteers aged 30.38 ± 2.85 years of which 13 females aged 31.5 ± 3.6 years and 9 males aged 29.25 ± 3.0 years. From the mentioned above group 16 volunteers agreed to undergo testing with all 3 devices (namely syringe–based device, 60 Pound BASELINE Algometer and AMF-500 Digital Force gauge) while all 22 agreed to undergo testing with syringe–based device and AMF-500 Digital Force gauge). Individuals reporting active pain, either acute or chronic, were excluded. All tests were conducted in a calm environment, with only the participant and the researcher present. Each above-mentioned device was applied 3 times at the midpoint of the forearm with 3 min-long interval between measurements. The same procedure was also repeated for the upper back (corresponding to the trapezius muscle), midpoint of the thigh region (corresponding to the quadriceps muscle) and plantar aspect of the foot.

In addition, we tested whether our device measurements were consistent with an existing device (AMF-500 Digital Force gauge) (13). We chose the anterior midpoint of the forearm, the trapezius muscle, the anterior midpoint of the thigh, and the sole of the foot (Figure 1A). The AMF-500 Digital Force gauge was applied to each point once with an interval of 1 min between applications. After a 3 min-long refractory period, the same test was repeated with the syringe-based algometer. Peak values of applied force at each pressure point were registered.

Concurrent validity of pressure and force measurements was estimated using linear regression utilizing SigmaPlot 11 for Windows (Grafiti LLC, USA). To assess data bias, we used Bland-Altman plots (11). Data correlation analysis was conducted utilizing Pearson correlation coefficient. For normality estimation we used Shapiro–Wilk normality test.

All experiments involving human subjects were approved by the Ariel University Medical School Ethical Committee (approval number AU-MED-DY-20231219).

## Results

**Figure 3:**
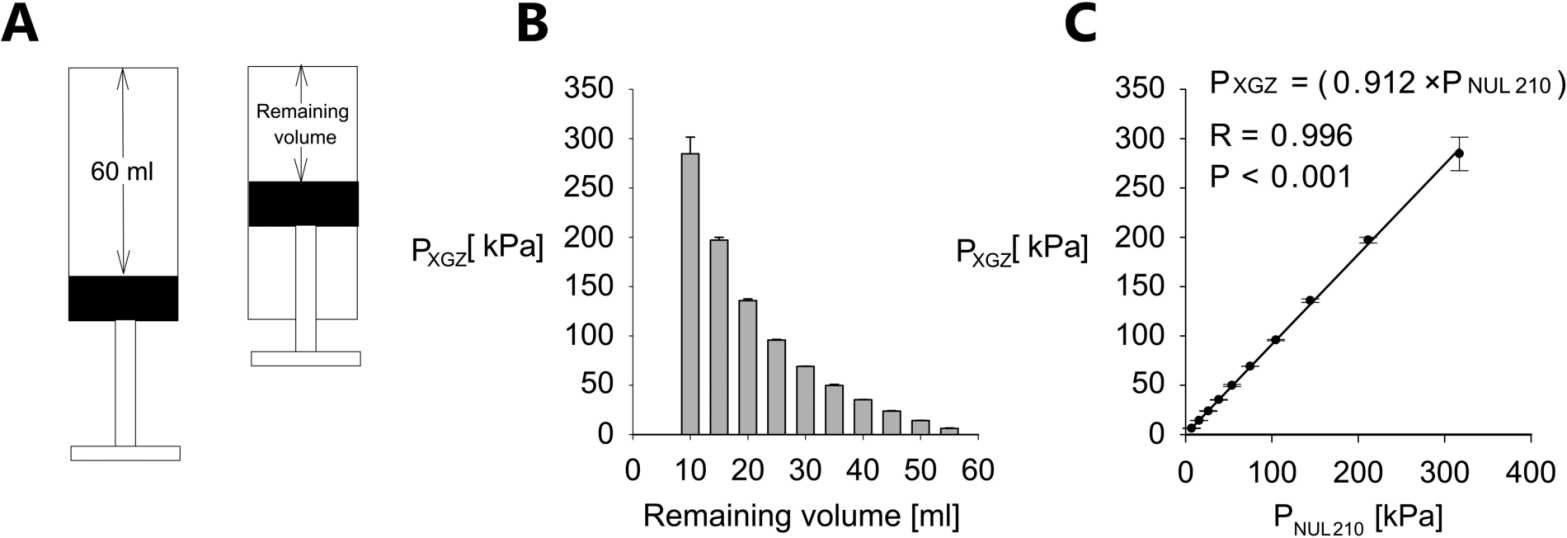
Pressure measurement validation. **(A)** Cartoon presentation of pressure measurement procedure. **(B)** P_XGZ_ (mean value ± standard error, *n* = 5, atmospheric pressure subtracted) vs. remaining volume for a 60 ml syringe. **(C)** P_XGZ_ vs. P_NUL210_ (NUL-210 pressure sensor, atmospheric pressure subtracted). Data shown as mean value ± standard error, *n* = 5 for each remaining volume. Error bars represent standard error. The correlation is assessed utilizing R—Pearson correlation coefficient, P—*P*-Value.

Initially we measured the inside syringe pressure (P_XGZ,_ atmospheric pressure subtracted) at various positions of the plunger corresponding to different remaining air volumes in syringe utilizing XGZP6847A sensor and compared the results to the NUL-210 pressure sensor (P_NUL210_, atmospheric pressure subtracted). Data obtained using a 60 ml syringe is shown in Figure 3. For each measurement the plunger was placed at the most extended position corresponding to remaining volume of 60 ml (Figure 3A). For each remaining volume pressure measurement was repeated 5 times (*n* = 5).

Considering the fact that several pressure ranges may be required for pressure threshold assessment of different patients and hand size of an examiner may vary too we conducted similar experiment for other syringes (3, 5 and 20 ml). Relevant data is summarized in Supplementary Materials Supplementary Figure S2s. In particular, for each of the above mentioned syringes generated pressure was measured for range of remaining air volumes in syringe and correlation between pressure measured for each volume was assessed between the XGZP6847A sensor and NUL-210 pressure sensor. Similar to the experiment with 60 ml syringe the plunger was placed at the maximal volume position at the beginning of each measurement. It can be seen from Supplementary Figure S2s that similar to data demonstrated in Figure 3 for 60 ml syringe there is a decremental relationship between the remaining volume and the pressure generated in the syringe (i.e., the lower the remaining volume the higher is the generated pressure) and also there is good correlation between the force generated by the plunger and the pressure measured inside the syringe for all tested syringes (3, 5 and 20 ml total volume) and whole range of the tested remaining volumes.

**Figure 4:**
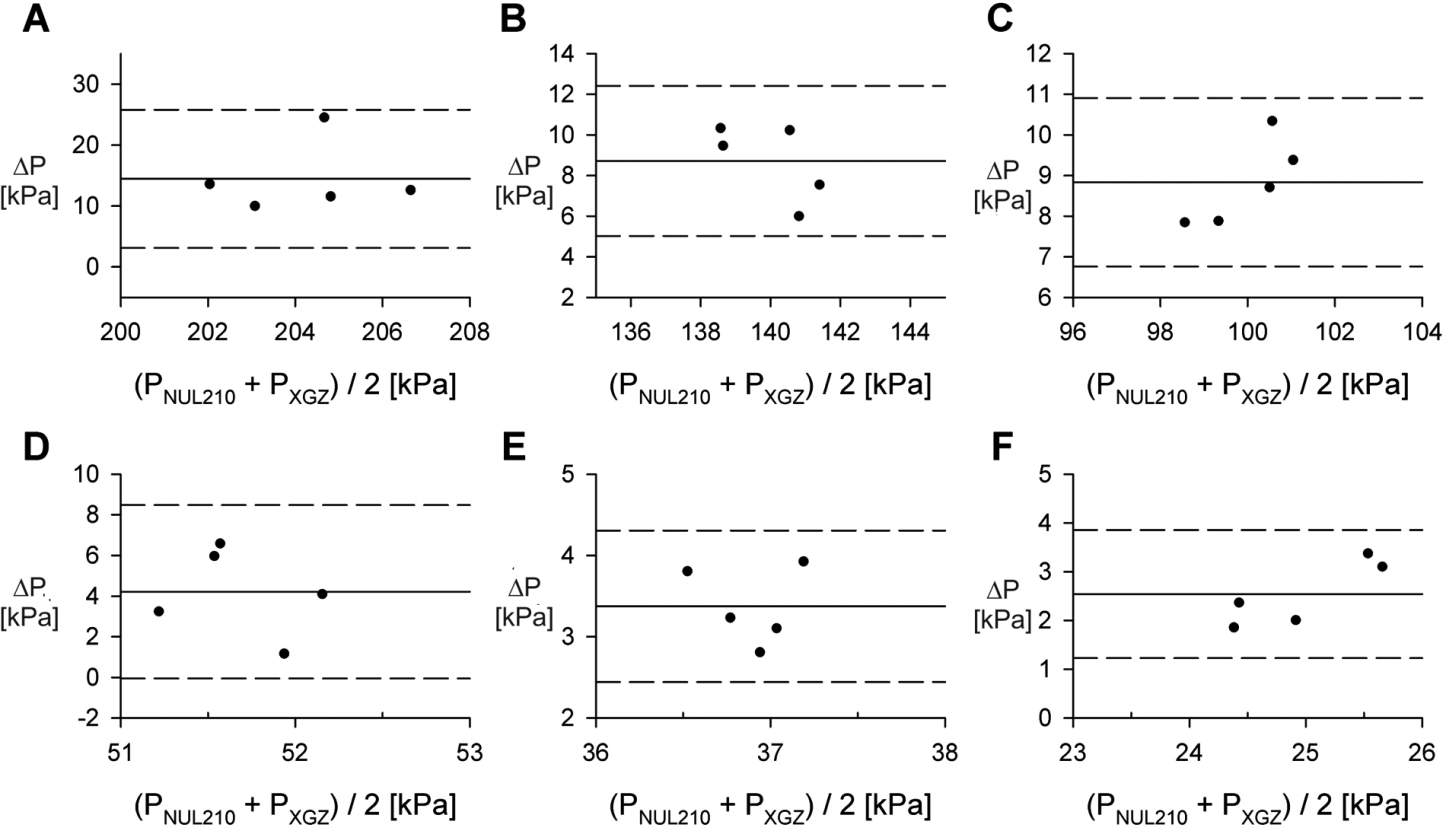
Comparison of the NUL-210 and XGZP6847A pressure sensors. **(A–F)** Bland-Altman plots comparing measurements of internal air pressure made with NUL-210 (P_NUL210_) and XGZP6847A (P_XGZ_) pressure sensors at specific volumes. Data obtained for 60 ml syringe. Each data point represents a separate measurement. Remaining volumes for corresponding plots: **(A)** 15 ml; **(B)** 20 ml; **(C)** 25 ml; **(D)** 35 ml; **(E)** 40 ml; **(F)** 45 ml. ΔP = P_NUL210_ - P_XGZ_.

To assess the possible bias of pressure measurements, we utilized Bland-Altman analysis for each remaining volume. Data is shown in Figure 4 and represents measurements conducted for 60 ml syringe.

Algometry data is commonly reported in units of force (12). We estimated the generated force for each pressure measurement based on Boyle's Law (14). We assumed that plunger tip is a cone and calculated its area utilizing an equation shown in Figure 2A. The base of the cone was assumed to be circle with diameter [equal to 2 radii (r)] measured utilizing an electronic caliper (plunger tip areas for different syringes are summarized in Table 1). We compared the calculated force values to those directly measured utilizing the NUL-225 force plate sensor. Collected data is summarized in Figures 2B–H.

**Table 1:** Surface area of rubber plunger lining (S on Figure 2A).

Syringe total volume (ml)	Lateral surface area of plunger lining [m^2^]
3	0.7·10^−4^
5	1.36·10^−4^
10	1.86·10^−4^
20	3.28·10^−4^
60	6.34·10^−4^

**Figure 5:**
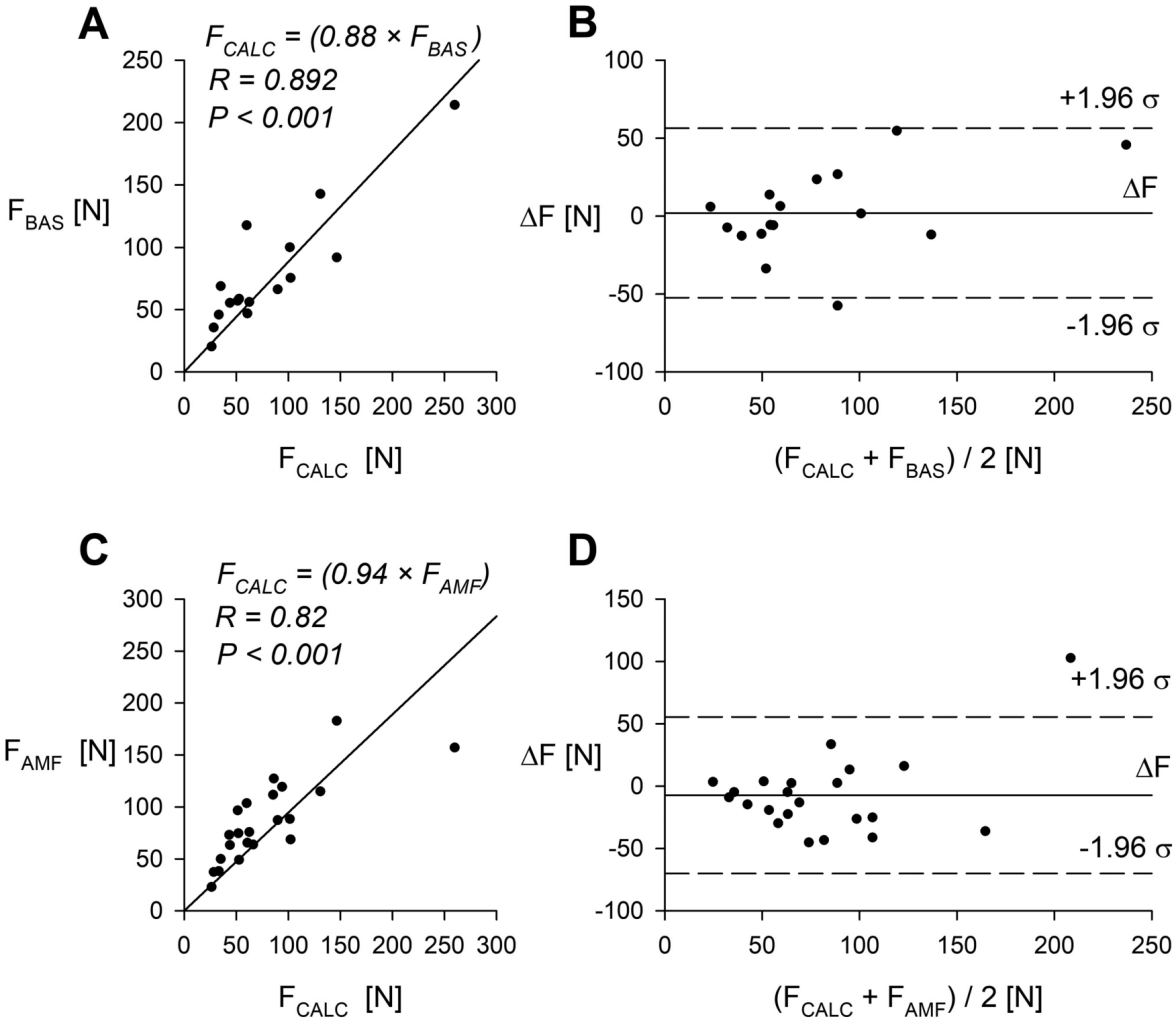
Comparison of the syringe-based algometer (60 ml) and two commercially available algometry devices. **(A)** Pain threshold measured with the syringe-based algometer and the BASELINE mechanical algometer (*n* = 16 volunteers). **(B)** Bland-Altman plot comparing the syringe-based algometer and the BASELINE algometer. **(C)** Pain threshold measured with the syringe-based algometer and the AMF-500 Digital Force gauge (*n* = 22 volunteers). **(D)** Bland-Altman plot comparing the syringe-based algometer and the AMF-500 Digital Force gauge. R—Pearson correlation coefficient. Data was obtained from midpoint of the right forearm.

Subsequently, we compared the pressure threshold data obtained with our device to that measured with two commercial algometers. The comparison was done at midpoint of the right forearm. Data collected from a group of human volunteers is shown on Figure 5.

**Figure 6:**
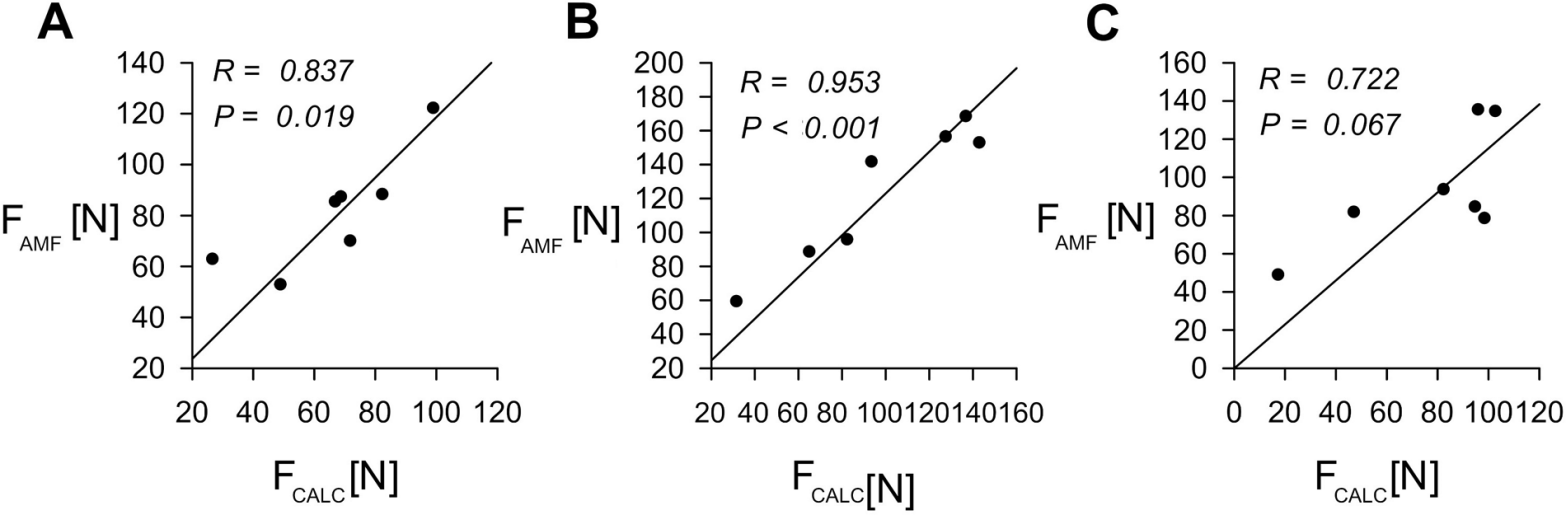
Comparison of the syringe-based algometer and the AMF-500 digital force gauge in commonly tested pressure points. **(A–C)** Comparison of force calculated from internal pressure readings (F_CALC_) and measured using the AMF-500 Digital Force gauge (F_AMF_). **(A)** at the trapezius muscle; **(B)** at the anterior midpoint of the thigh; **(C)** at the sole of the foot. See Figure 1A for specific locations. R—Pearson correlation coefficient, P—*P*-Value.

In addition, we compared measurements taken with the syringe-based algometer and with the AMF-500 Digital Force gauge over another 3 commonly tested pressure spots. Results and comparison are presented in Figure 6.

## Discussion

In this study, we present a low-cost algometer constructed from a plastic syringe, a pressure sensor and an Arduino microcontroller. The performance of this device was extensively evaluated utilizing the Nul-210 pressure sensor and the Nul-225 force sensor. In both cases, correlation coefficients obtained from linear regression analysis were close to 1, as demonstrated in Figures 3B, 2B, indicating good concurrent validity with 2 sensors (Nul-210 and Nul-225). Moreover, our device demonstrated high precision in internal pressure measurements, with a standard error-to-signal ratio of 0.1%–3.4% (Figure 3A).

Blind-Altman analysis of pressure measurement (Figure 4) comparing data obtained utilizing Nul-210 pressure sensor and XGZP6847A sensor demonstrated <15% absolute difference between both sensors for all remaining volumes measured for 60 ml syringe. It must be emphasized that we observed positive differences between measurements generated by both sensors. This finding may indicate bias introduced by different physical characteristics of both sensors (in particular radius of nozzle of Nul-210 sensor is 0. 65 mm and of XGZP6847A sensor is 0.25 mm). Based on these findings, we suggest that our device exhibits acceptable concurrent validity and precision compared to other pressure measuring devices.

Direct comparison of pain threshold measurements utilizing our device with those obtained from two commercially available algometers demonstrated good agreement when tested on human volunteers midpoint forearm (Figures 5A,C) and for another three common pain threshold testing points (Figure 6).

Several digital algometers are currently available on the market, including the Algomed (Medok Instruments, Israel), Commander Echo (MTM, Canada), Wagner FPIX (Wagener Instruments, USA) among others. The cost of these devices is typically ranging from 900 USD to several thousand USD, making them financially prohibitive for newer or smaller laboratories (15). In contrast, the total cost of our device, including the sensor, syringe, connectors and tubing—remains below $150. As of now, all components are readily available for purchase internationally.

Syringe-based algometers were described in previous studies, such as for short-term use in emergency response units (10) or for examination of coccydinia (15). Major advantages of our device is integration of an analog pressure sensor and Arduino microcontroller. This combination provides reasonable solution to calibration challenge inherent to earlier syringe-based algometers (10). Additionally, high frequency data sampling (up to 15 kHz) and multi-channel data acquisition become available. It can be seen from examination of the attached script, that required programming skills are minimal (see Supplementary Material). Moreover, in the future the multi-channel acquisition capability of our device could be expanded to connect several biometric sensors (*e.g.,* electrodermal activity, ECG, EMG) to the same microcontroller with lower cost than previously described devices. Furthermore, our device can be modified in order to measure not only pain pressure threshold but also pressure tolerance. This feature will require either improvement of our device ergonomic properties (i.e., adding handle) or further development (including automatization of force application).

In summary, current study describes an affordable, low cost, and high precision algometry device designed for measuring Pain Pressure threshold and Pain tolerance. With future development and necessary certification, this device has the potential to be implemented as a medical device in the future.

## Data Availability

The original contributions presented in the study are included in the article/Supplementary Material, further inquiries can be directed to the corresponding author.

## References

[1] Andrews P Steultjens M Riskowski J. Chronic widespread pain prevalence in the general population: a systematic review. Eur J Pain. (2018) 22(1):5–18. 10.1002/ejp.1090 28815801

[2] Hoy D Bain C Williams G March L Brooks P Blyth F A systematic review of the global prevalence of low back pain. Arthritis Rheum. (2012) 64(6):2028–37. 10.1002/art.34347 22231424

[3] Pak DJ Yong RJ Kaye AD Urman RD. Chronification of pain: mechanisms, current understanding, and clinical implications. Curr Pain Headache Rep. (2018) 22(2):9. 10.1007/s11916-018-0666-8 29404791

[4] Breivik H Borchgrevink PC Allen SM Rosseland LA Romundstad L Hals EKB Assessment of pain. Br J Anaesth. (2008) 101(1):17–24. 10.1093/bja/aen103 18487245

[5] Carneiro AM de Góes Salvetti M Dale CS da Silva VA. Quantitative sensory testing in fibromyalgia syndrome: a scoping review. Biomedicines. (2025) 13(4):988. 10.3390/biomedicines13040988 40299678 PMC12025226

[6] Ahmad B Barkana BD. Pain and the brain: a systematic review of methods, EEG biomarkers, limitations, and future directions. Neurol Int. (2025) 17(4):46. 10.3390/neurolint17040046 40278417 PMC12029872

[7] Kamińska A Dalewski B Sobolewska E. The usefulness of the pressure algometer in the diagnosis and treatment of orofacial pain patients: a systematic review. Occup Ther Int. (2020) 2020:5168457. 10.1155/2020/5168457 32684869 PMC7341437

[8] Melia M Schmidt M Geissler B König J Krahn U Ottersbach HJ Measuring mechanical pain: the refinement and standardization of pressure pain threshold measurements. Behav Res. (2015) 47(1):216–27. 10.3758/s13428-014-0453-3 24570335

[9] Kinser AM Sands WA Stone MH. Reliability and validity of a pressure algometer. J Strength Cond Res. (2009) 23(1):312–4. 10.1519/JSC.0b013e31818f051c 19130648

[10] Johnson TW Watson PJ. An inexpensive, self-assembly pressure algometer. Anaesthesia. (1997) 52(11):1070–2. 10.1111/j.1365-2044.1997.226-az0361.x 9404169

[11] Haghayegh S Kang H-A Khoshnevis S Smolensky MH Diller KR. A comprehensive guideline for Bland-Altman and intra class correlation calculations to properly compare two methods of measurement and interpret findings. Physiol Meas. (2020) 41(5):055012. 10.1088/1361-6579/ab86d6 32252039

[12] Fischer AA. Pressure algometry over normal muscles. Standard values, validity and reproducibility of pressure threshold. Pain. (1987) 30(1):115–26. 10.1016/0304-3959(87)90089-3 3614975

[13] Passmore SR Descarreaux M. Performance based objective outcome measures and spinal manipulation. J Electromyogr Kinesiol. (2012) 22(5):697–707. 10.1016/j.jelekin.2012.02.005 22406070

[14] Hobbie RK Roth BJ. Intermediate Physics for Medicine and Biology. New York, NY: Springer (2007).

[15] Mohanty PP Pattnaik M. Effect of stretching of piriformis and iliopsoas in coccydynia. J Bodyw Mov Ther. (2017) 21(3):743–6. 10.1016/j.jbmt.2017.03.024 28750995

