# Supplementary material for: A syringe-based digital algometer with a USB interface: a low-cost alternative to commercially available devices
Source: Front Pain Res (Lausanne). 2025 Sep 4;6:1652241. doi: 10.3389/fpain.2025.1652241 (PMC12443797; doi:10.3389/fpain.2025.1652241)
Supplement: Supplementary file 1 [file Supplementaryfile1.pdf]

Supplementary Materials

Table 1s. Utilized sensors and algometry devices	
Sensor	Specifications
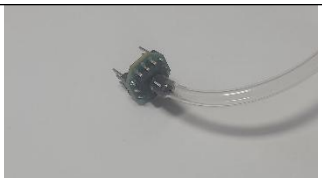<br>XGZP6847A Analog Pressure Sensor	Range: 0 kPa ~ 1000 kPa<br>ADC Resolution: 10 bit
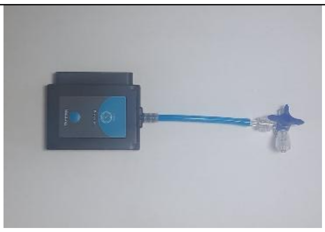<br>NeuLog NUL-210 Pressure Sensor	Range: 0 kPa ~ 700 kPa<br>ADC Resolution: 16 bit<br>Resolution: 0.1 kPa
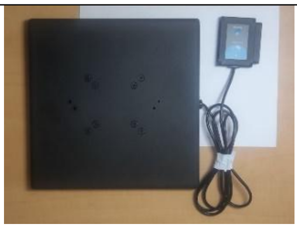<br>NeuLog NUL-225 Force Plate Sensor	Range: -800 N ~ 2000 N<br>ADC Resolution: 16 bit<br>Resolution: 1 N
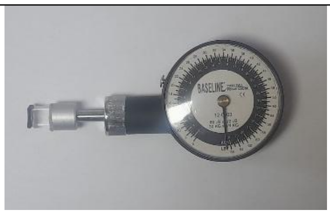<br>BASELINE 12-1443 Algometer	Range: 0 N ~ 294 N
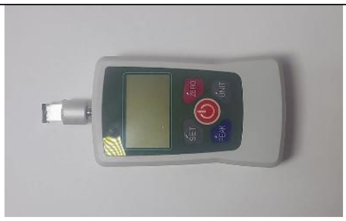<br>AMF-500 Force Gauge	Range: 0 N ~ 500 N<br>Resolution: 0.1 N

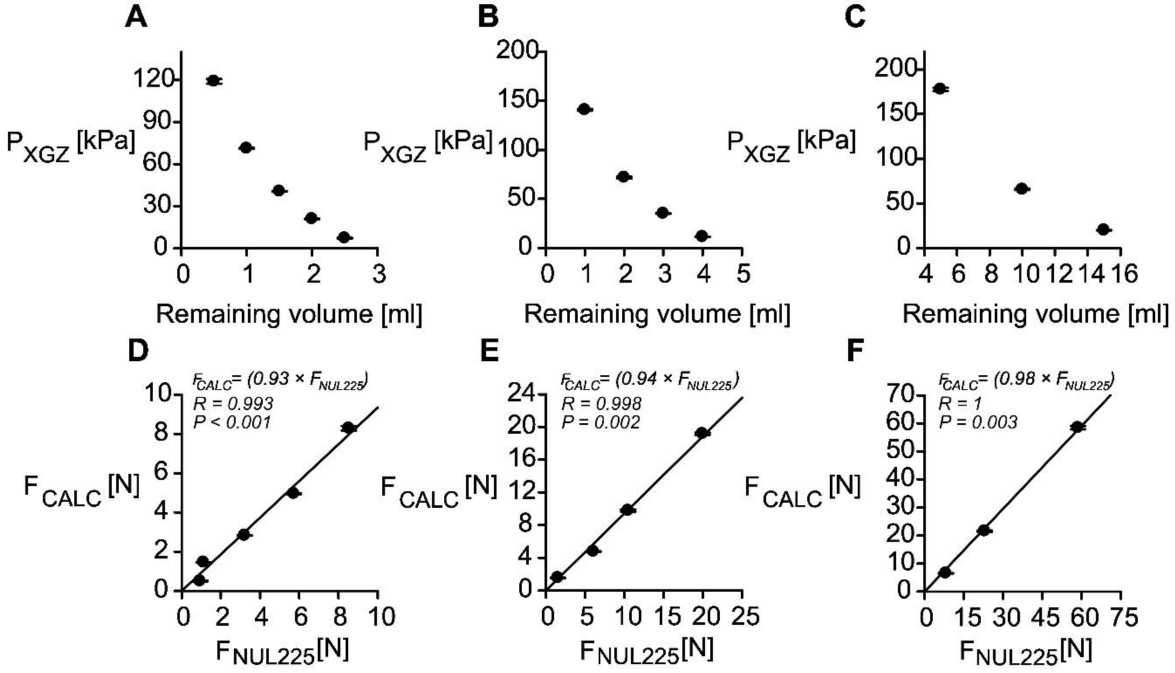

Figure 2s. Precision of the syringe-based algometer (3ml, 5ml, 20ml) in repeated tests. A-D. Averaged XGZP6847A pressure sensor measurements at specific volumes. **A:** 3 ml; **B:** 5 ml; **C:** 20 ml. **D-F.** Linear relationship between expected force values calculated from measurements of internal pressure and force applied to a horizontal surface of the NUL-225 force plate. **D:** 3 ml; **E:** 5 ml; **F:** 20 ml.

**Script for data acquisition using the Arduino Microcontroller**
<pre>int psa = A1;<br>void setup() {<br>pinMode (psa, INPUT);<br>Serial.begin(9600);<br>}<br>void loop() {<br>int data= analogRead(psa); //raw sensor output<br>float P = 1e3*(data-100)/1024; //pressure, KPa<br>Serial.print(P); //print pressure (kPa)<br>Serial.println(",");<br>// Serial.println(millis()); //print timestamp, ms (optional)<br>}</pre>

The script was designed to calculate the internal air pressure in kPa using **Eq. 1:**

$$P = 10^3 \times \frac{data - 100}{1024},$$

Where *P* – internal air pressure (kPa), *data* – 10-bit output of the sensor. *100* – raw output of the sensor in atmospheric pressure.

The *P* variable is printed at the end of every cycle into a separate, comma-delineated column of a text file.

The script includes an optional line that prints a timestamp relative to the start of acquisition (greyed out, remove "//" to activate).

To convert the values of pressure (Kpa) into applied force (N) we utilized **Eq. 2**:

$$F = P \times 10^3 \times S,$$

where F - calculated force (N), P – internal pressure value (kPa), S - the surface area of the pressure probe (m^2). Surface areas for probes of different syringes are listed in **Table 1.**
